# Effect of limb surrogate surface compliance on the impact response of wrist protectors

**DOI:** 10.1016/j.jsampl.2023.100023

**Published:** 2023-04-27

**Authors:** Gemma E. Leslie, Keith Winwood, Weizhuo Wang, Nick Hamilton, Tom Allen

**Affiliations:** a Musculoskeletal Science and Sports Medicine, Manchester Metropolitan University, Manchester, M1 5GD, UK; b Advanced Materials and Surface Engineering, Manchester Metropolitan University, Manchester, M1 5GD, UK; c Sports Engineering Research Group, Sheffield Hallam University, Sheffield, S10 2LX, UK

**Keywords:** Impact test, Wrist surrogate, Standard, Certification, Sporting PPE

## Abstract

**Objectives:**

To investigate the effect on the impact response of wrist protectors by incorporating a soft tissue simulant on to a wrist surrogate made of stiff material. The effect of protector strapping condition was also investigated.

**Design and methods:**

A compliant and a stiff surrogate were made, based on the wrist geometry specified in ISO 20320:2020 for the “Limitation of wrist extension” test. Two styles of wrist protectors (short, long) were tested on each limb surrogate, subject to a ∼31 ​J impact. Six protectors of each style were tested, with two of each at each strapping condition (loose, moderate, tight) on each surrogate (stiff, compliant) (24 combinations). Example temporal force and wrist angle and force vs. wrist angle plots are presented for comparison between conditions.

**Results:**

When protectors were on the compliant surrogate, peak impact force was 55–68% lower (short 3.1 vs. 6.8 ​kN, long 2.7 vs. 8.3 ​kN). The time to reach this peak force was ∼4 ​ms (12%) longer, than for the stiff surrogate. Protector strapping condition had no clear effect for the stiff surrogate, with the wrist extending to its limit for all tests. Strapping protectors tighter on the compliant surrogate tended to decrease the maximum wrist angle and peak force.

**Conclusions:**

With results being sensitive to surrogate design and strapping condition, these both need to be clearly reported in future work impact testing wrist protectors, with implications for certification tests within standards.

## 1 Introduction

Certification tests for sporting personal protective equipment (sPPE) typically utilise a basic geometric human limb surrogate or anvil, often made of a stiff material [1]. Following a call from sports safety experts [2], a standard for snowboarding wrist protectors was published as ISO 20320:2020 [3]. This standard includes a quasi-static bend test (Limitation of wrist extension) and a drop-tower style impact test (Impact performance). A wrist surrogate made from polyamide or similar material and a steel hemispherical anvil are prescribed for these respective bend and impact tests. The simplicity and robustness of such surrogates and anvils makes them suitable for certification tests performed in test houses, where repeatability is crucial. There could be benefits from having more representative human limb surrogates for developing and testing sPPE [1,[4], [5], [6]], which are also simple to reproduce, robust and offer a repeatable response.

Surrogate shape has been shown to influence the measured stiffness of wrist protectors in a quasi-static bend test [7]. To represent skin, Leslie et al. [8] incorporated a layer of silicone to the surrogate design specified in ISO 20320:2020 for the bend test. Protectors fitted to the modified surrogate had higher stiffness measurements than when on the original design. Furthermore, as expected [8,9], strapping protectors more tightly increased the measured stiffness. The effect of surrogate design and protector strapping condition has not been studied in impact testing.

The ISO 20320:2020 impact test [3] only assesses the cushioning properties of the palmar region of a protector, and the hemispherical anvil is not wrist shaped [10,11]. This impact test is like the one in EN 14120:2003 (roller sports wrist protectors) [12], and it is likely that simplicity and repeatability were prioritised when these tests were developed. Adams et al. [10] developed a more holistic test for impacting a protector utilising a wrist surrogate based on 3D data of an arm. This test allows simultaneous assessment of how well a protector reduces impact force and limits wrist extension. There is scope to develop the work of Adams et al. [10], with a view to incorporating a wrist surrogate impact test into standards for wrist protectors, including ISO 20320:2020 and EN 14120:2003.

Matching the surrogate geometry in an impact test to the one in ISO 20320 would allow, i) it to be reproduced from a schematic diagram and ii) more meaningful comparisons between these two standardised tests [11]. Furthermore, incorporating a soft tissue simulant, as published by previous authors when developing limb surrogates [1,[4], [5], [6],^8^,^13^,^14^], should make the surrogate more representative of a human arm than one fabricated of a stiff material used by Adams et al. [10]. Based on higher stiffness measurements in bend testing [8,9], we hypothesise that both adding an outer layer of silicone to the surrogate and increasing protector strapping tightness would reduce impact forces and wrist angles. A geometric shaped wrist surrogate was developed for impact testing. The effect of introducing a compliant outer layer to this surrogate on the impact response of wrist protectors, across different strapping conditions, was investigated.

## 2 Methods

### 2.1 Surrogate design and fabrication

**Fig. 1:**
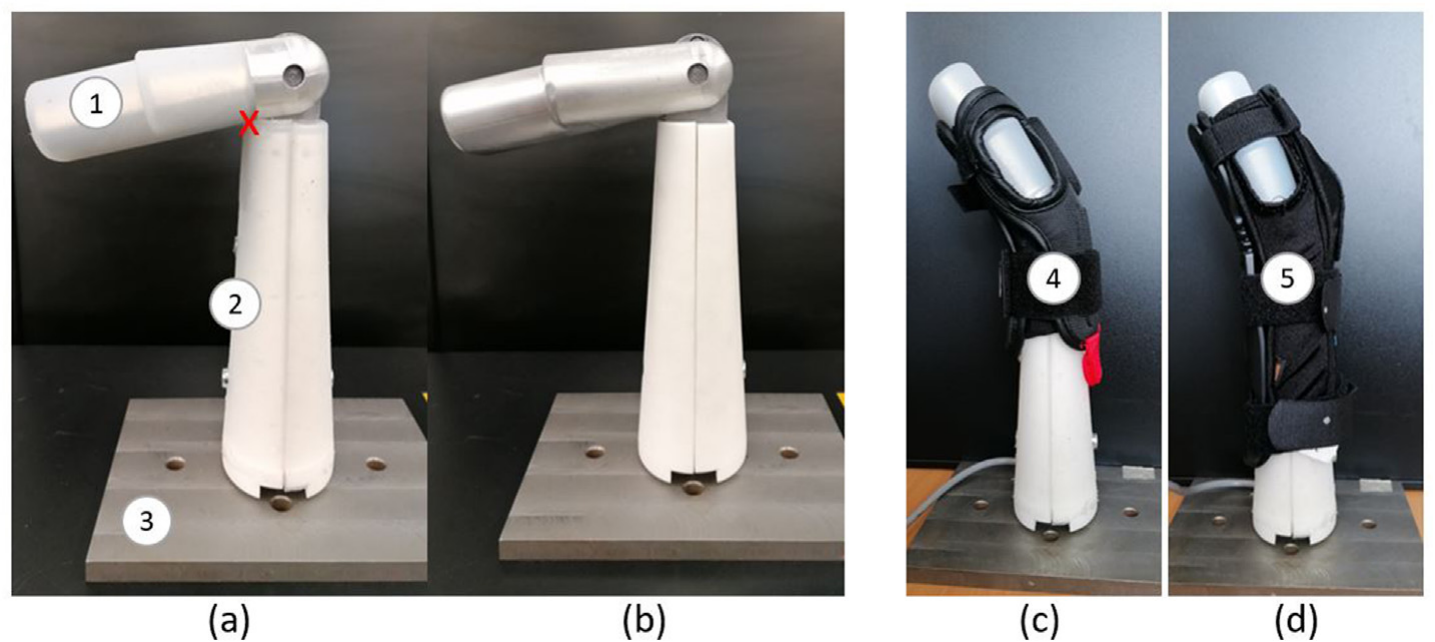
The (a) compliant and (b) stiff wrist surrogate at their wrist extension limit at rest (unprotected condition), and the compliant surrogate fitted with the (c) short and (d) long protector. 1 – hand, 2 – forearm casings, 3 – base plate, 4 – short protector, 5 – long protector. The red cross on (a) indicates the region of silicone which can compress when the hand is forced backwards, causing a higher maximum wrist extension limit. Note the difference in hand position between the unprotected (a, b) and protected (c, d) surrogate – full extended vs. raised.

Based on the design of Adams et al. [10], details of the surrogates used are in Online Resource 1 Section 1 and summarised here. The surrogates were based on the medium sized geometry specified in ISO 20320:2020 (Section 5.8.2). They consisted of two parts: interchangeable hand and two forearm casings fitted to a universal central core (Fig. 1). The two sets of interchangeable surrogate parts were: one with an outer layer of silicone (compliant, Fig. 1a) and the other without (stiff, Fig. 1b). The silicone was a maxillofacial silicone M511 (Technovent, Bridgend, UK) as previously reported [8]. M511 is commonly used within facial prosthetic rehabilitation and was used by Payne et al. [1] to replicate soft tissue in a limb surrogate for testing sPPE. The hardness of the silicone was measured at four locations on the surrogate palm, both before and after impact testing, using a Shore durometer hardness type A-2 (The Shore Instrument & MFG Co, New York, USA). The mean values for the Shore A hardness measurements taken before and after impact were both 26, with respective standard deviations of 2.0 and 1.0.

The silicone layer was 7 ​mm thick on the palmar side of the hand [15], with 3 ​mm thickness elsewhere on the hand and on the forearm casings (Fig. 1a) [8]. The stiff hand and forearm casings were the same external size and shape as their compliant counterparts, including the space occupied by the silicone (Fig. 1b). Based on the geometry specified in ISO 20320:2020, both surrogates had a wrist extension limit of ∼103° at rest (Fig. 1a and b). As an artefact of the silicone near the wrist joint, the hand of the compliant surrogate extended further when loaded, from ∼103° to ∼115° (Fig. 1a).

### 2.2 Impact rig

The pendulum impact rig from Adams et al. [10] was used, with some modifications. These included reducing the effective striking mass (from ∼10 to ∼6.5 ​kg) so a wider range of impact energies could be achieved while maintaining a sufficiently high release height and hence impact speed. The thickness of the aluminium plate used for mounting polychloroprene blocks on the impactor (for tuning loading response) was reduced (from 4 to 1 ​cm, ∼2.5 to ∼0.6 ​kg), as well as the number of blocks (from 5 to 2, ∼2.6 to ∼1 ​kg). Two synchronised high-speed cameras (Phantom® Micro R110, Vision Research UK), fitted with a zoom lens (Nikon AF Nikkor 24–85 ​mm 1:2.8–4 D, Nikon Corporation, Japan), filmed the impact (10 ​kHz, 320 ​× ​400 pixels). One camera was positioned side-on to measure wrist angle (see Online Resource 1 Section 2), with the other viewing the dorsal side of the protector. A dynamometer was placed under the surrogate base place to measure impact force, with a sampling frequency of 20 ​kHz [10]. The cameras and dynamometer were synchronised with a trigger and Data Acquisition Device (DAQ).

### 2.3 Test method

A cadaveric arm fracture load range based on values summarised by Adams et al. [10] was used to gauge impact severity (mean and standard deviation of 2.7 ​± ​0.8 ​kN, ranging from ∼1 to 4 ​kN). The pendulum release height was 0.5 ​m (to top of central core), corresponding to an impact energy of ∼31 ​J. The hand of the protected surrogates was set to a start angle of ∼35° in extension. A maximum non-injurious wrist extension angle of 85°, from six studies [[16], [17], [18], [19], [20], [21]] (see Online Resource 1 Section 3), was used to gauge the ability of protectors to prevent wrist hyperextension. The maximum wrist extension angles from these studies were comparable to those reported in Greenwald et al. [22] for non-injurious snowboarding falls.

Two styles of snowboarding wrist protectors matching those used in prior work [[7], [8], [9], [10], [11]] (both new, adult medium, left hand), were tested (“short protector” - Burton© and “long protector” - Flexmeter™ double sided). The “skid plate” on the palm of the long protectors was removed before testing.^1^ The test conditions (surrogate-protector-strapping) matched those of Leslie et al. [8]. Six protectors of each style (short, long) were tested, with two of each tested at three strapping conditions (loose, moderate, tight) on each surrogate (stiff, compliant), creating 24 different combinations. Using a method developed by Adams et al. [9], the strapping condition's related to a 1, 2 or 3 ​kg (loose, moderate and tight respectively) mass hung from the straps, before rotating the surrogate to secure the straps, with the resulting position marked for future reference [8,9]. Three repeated tests were performed for each surrogate-protector-strapping combination (total of 72 tests), with 15-min rest between them.

Testing was conducted over two days, with 36 tests on protected surrogates each day. Room temperature was recorded before and after testing on each day (range 21.5–21.8 ​°C). Protectors were defined as either: new (untested) or used (after three impacts). Surrogates were alternated between combinations, so a new protector of each style was tested at each strapping condition on each surrogate. Protectors were re-positioned and re-strapped between tests. The pair of polychloroprene blocks on the impactor was changed after every 24 impacts on a protected surrogate. Unprotected impacts were conducted for each of the three pairs of blocks: one before testing protectors to “condition” the polychloroprene, and three during testing to monitor its response (after 8, 16 and 24 impacts on a protected surrogate), and for comparison with a protected surrogate [10]. The coefficient of variation of the nine unprotected impacts (excluding the “conditioning impact” on each pair) across the three pairs of blocks was used to gauge the consistency of the polychloroprene response [10].

### 2.4 Data analysis

Vertical impact force was calculated from the output voltage of the dynamometer using the calibration factor from Adams et al. [10]. Force data was low-pass filtered (4-pole phaseless Butterworth digital filter) at Channel Frequency Class 1000 (1650 ​Hz cut-off frequency) in MATLAB® (vR2017a, MathWorks®, USA) [23]. Temporal force, temporal wrist angle and force vs. wrist angle were plotted, for the first impact at each strapping condition, with peak force aligned at time (t) ​= ​0 ​s for comparison between conditions. General linear model univariate analysis was performed using SPSS statistical software (IBM® SPSS® Statistics Premium 27) at a significance level of p ​< ​0.05 to determine the main effects for each surrogate individually [24]. Peak force was set as the dependant variable, and protector style, strapping condition and protector condition as the independent variables.

## 3 Results

**Fig. 2:**
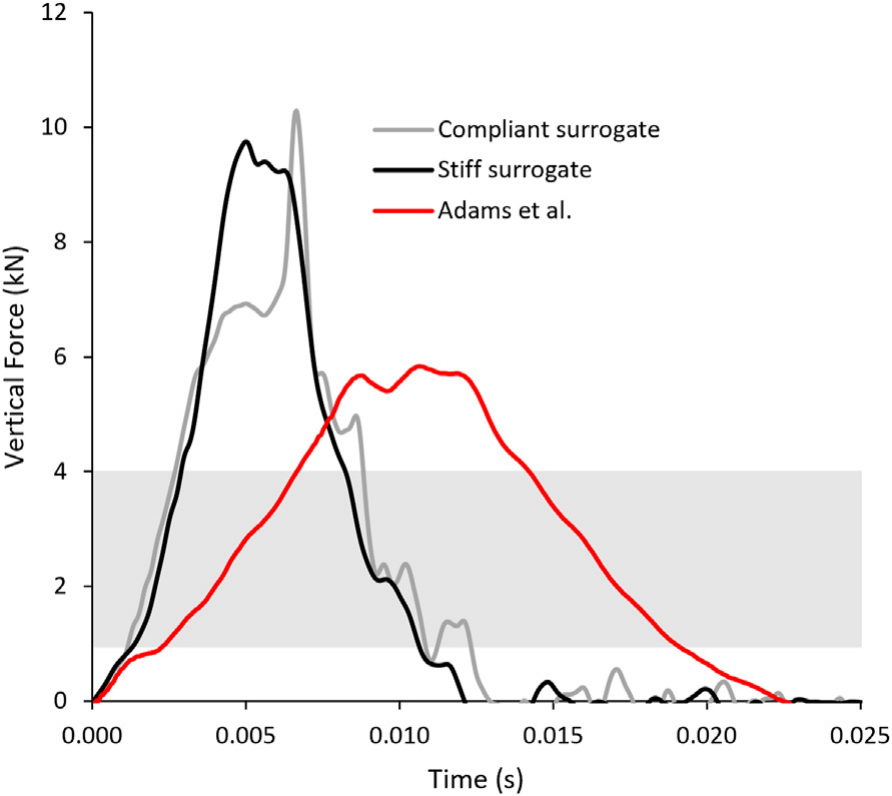
Temporal force plot showing the impact traces for the compliant and stiff surrogate from this study (drop height ∼0.5 ​m), compared to Adam's et al. [11] (presented in Fig. 3 of that paper) with a different surrogate, impactor and impact energy to those used here. The grey box highlights the cadaver fracture range of 1–4 ​kN.

The coefficient of variation for peak force of the unprotected impacts was <3%, similar to previous work [10]. An example temporal force trace for each unprotected surrogate is displayed in Fig. 2, alongside one of Adams et al. [10] and the cadaver fracture range (1–4 ​kN). The two new surrogates had similar initial loading rates and peak forces, with more discrepancy around the highest forces and during unloading. The loading rates and peak forces on these new surrogates were higher than that of Adams et al. [10], indicating the impact scenario presented here was more severe, with forces far above the cadaveric fracture range.

**Fig. 3:**
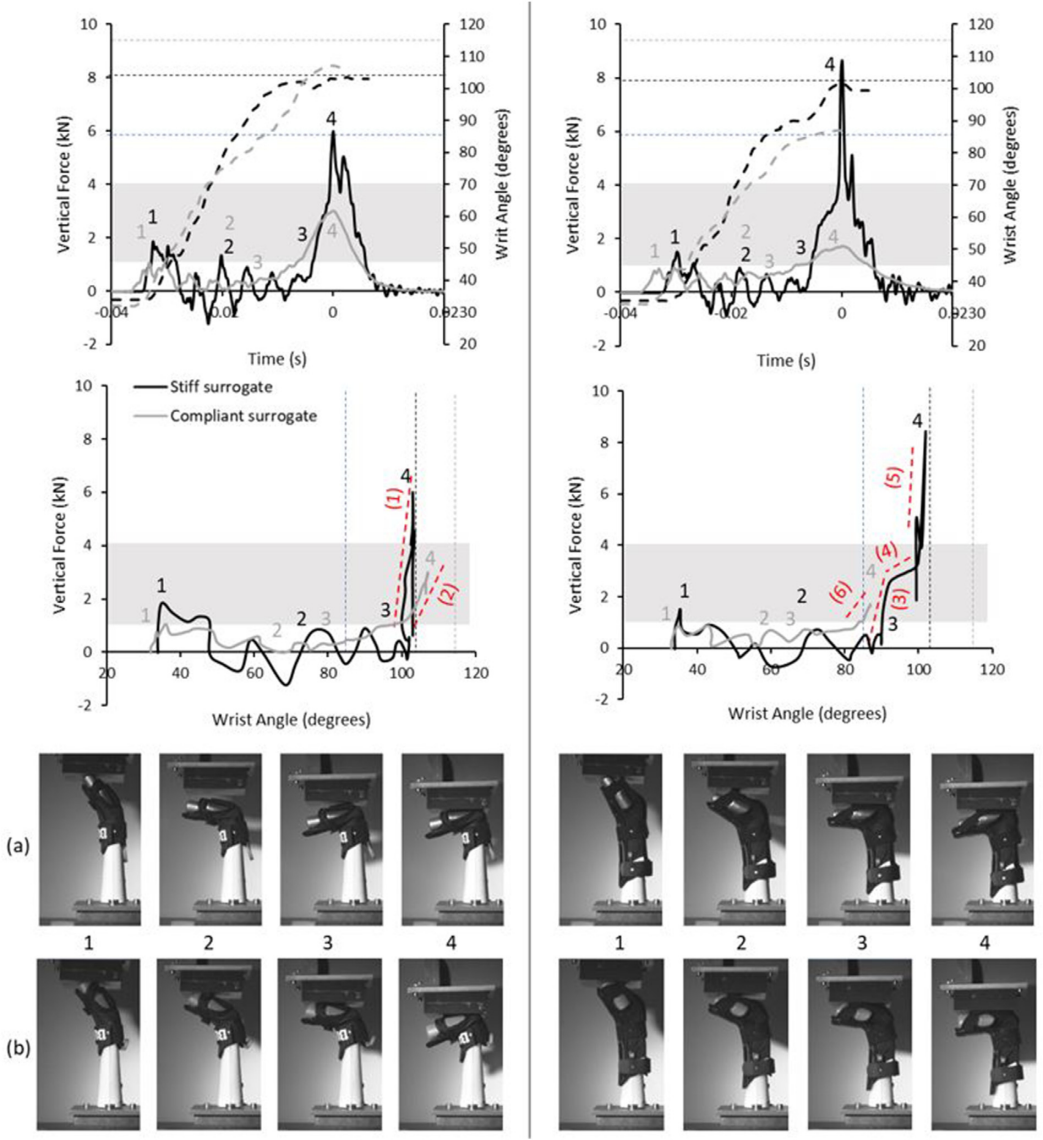
Temporal force and temporal wrist angle trace (top) and force vs. wrist angle (bottom) of the first impact of the short (left) and long protector (right) strapped at moderate condition on the stiff and compliant surrogate, alongside a sequence of high-speed images which showcase the key points when on the (a) stiff and (b) compliant surrogate. Dashed curves on the top graph indicate the wrist angle. The grey box highlights the cadaver fracture range (∼1–4 ​kN). The dashed straight lines indicate the reported non-injurious maximum wrist extension (85°), and the wrist extension limit of the surrogates (stiff ∼103°, compliant ∼115°). The red dashed trend lines indicate the gradient, where (1) is 1014 ​N/° and (2) is 301 ​N/°, (3) is 782 ​N/°, (4) is 97 ​N/°, (5) is 2009 ​N/° and (6) 225 ​N/°.

**Fig. 4:**
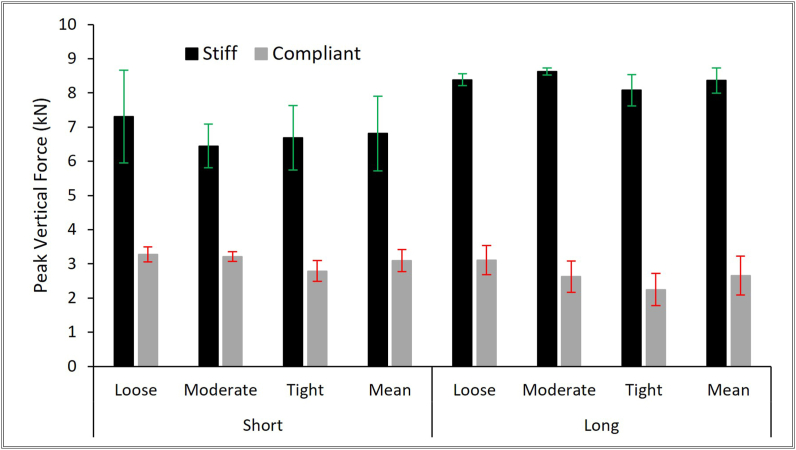
Mean peak force for each strapping condition for the short and long protectors (error bars showing mean ​± ​SD).

Example temporal force, temporal wrist angle and force vs. wrist angle traces for both protectors at moderate strapping condition on both surrogates are displayed in Fig. 3. The cadaver fracture range and the maximum non-injurious wrist extension (85°) are included, and the test data followed similar trends to results presented by Adams et al. [10]. For these examples, peak forces were lower for the compliant surrogate, as found for all tests (Fig. 4), while taking longer to reach peak force (∼2–4 ​ms, 6–12%).

The initial spike in force (Fig. 3 – Point 1 on graphs), due to the pendulum striking the uppermost part of the hand (Frame 1), was below the cadaver fracture range when either protector was fitted to the compliant surrogate, with the peak force (Point 4 on graphs) within that range. When the stiff surrogate was fitted with either protector, the initial force spike lay within the cadaver fracture range, whereas the peak force exceeded that range. Both surrogates exceeded the reported maximum non-injurious wrist extension angle (85°) when fitted with either protector. The stiff surrogate reached its wrist extension limit (∼103°) with either protector (Fig. 3). The compliant surrogate did not reach the resting wrist extension limit with the long protector, but it exceeded it with the short one (due to the silicone compressing), reaching ∼87° and ∼107° respectively (Fig. 3). Both protectors had a similar force-angle gradient to peak force when on the compliant surrogate (301 vs. 225 ​N/°). When on the stiff surrogate, the short protector had an almost constant steep gradient to peak force (1014 ​N/°), unlike the long protector, which had a three-part gradient to peak force (782, 97 and 2009 ​N/°) (Fig. 3).

**Fig. 5:**
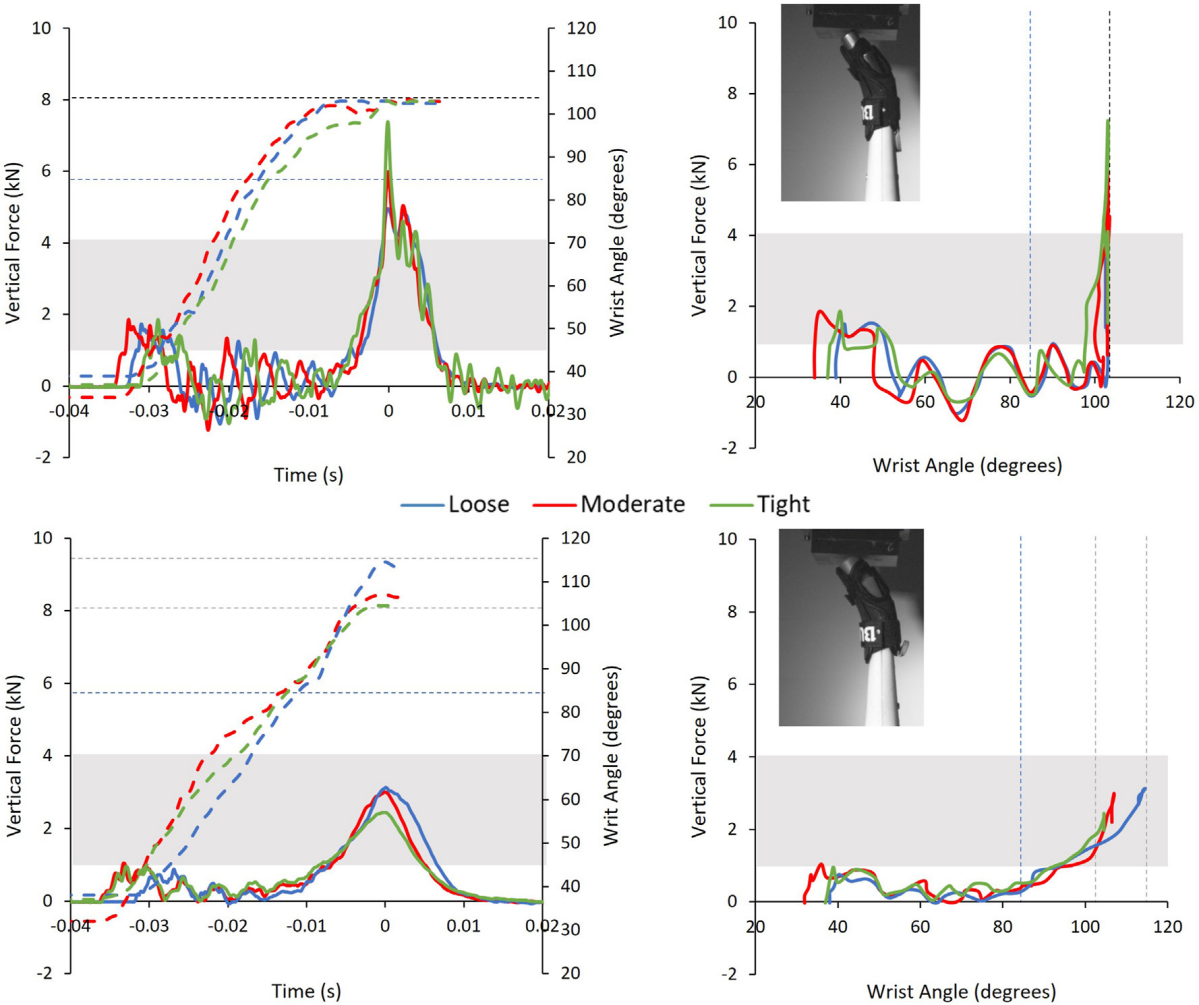
Temporal force and temporal wrist angle (left) and force vs. wrist angle (right) of the first impact of the short protector between strapping conditions on the stiff (top) and compliant surrogate (bottom). Dashed curves indicate the wrist angle. The grey box highlights the cadaver fracture range (∼1–4 ​kN). The dashed straight lines indicate the reported non-injurious maximum wrist extension (85°) and the wrist extension limit of the surrogates (stiff ∼103°, compliant ∼115°).

**Fig. 6:**
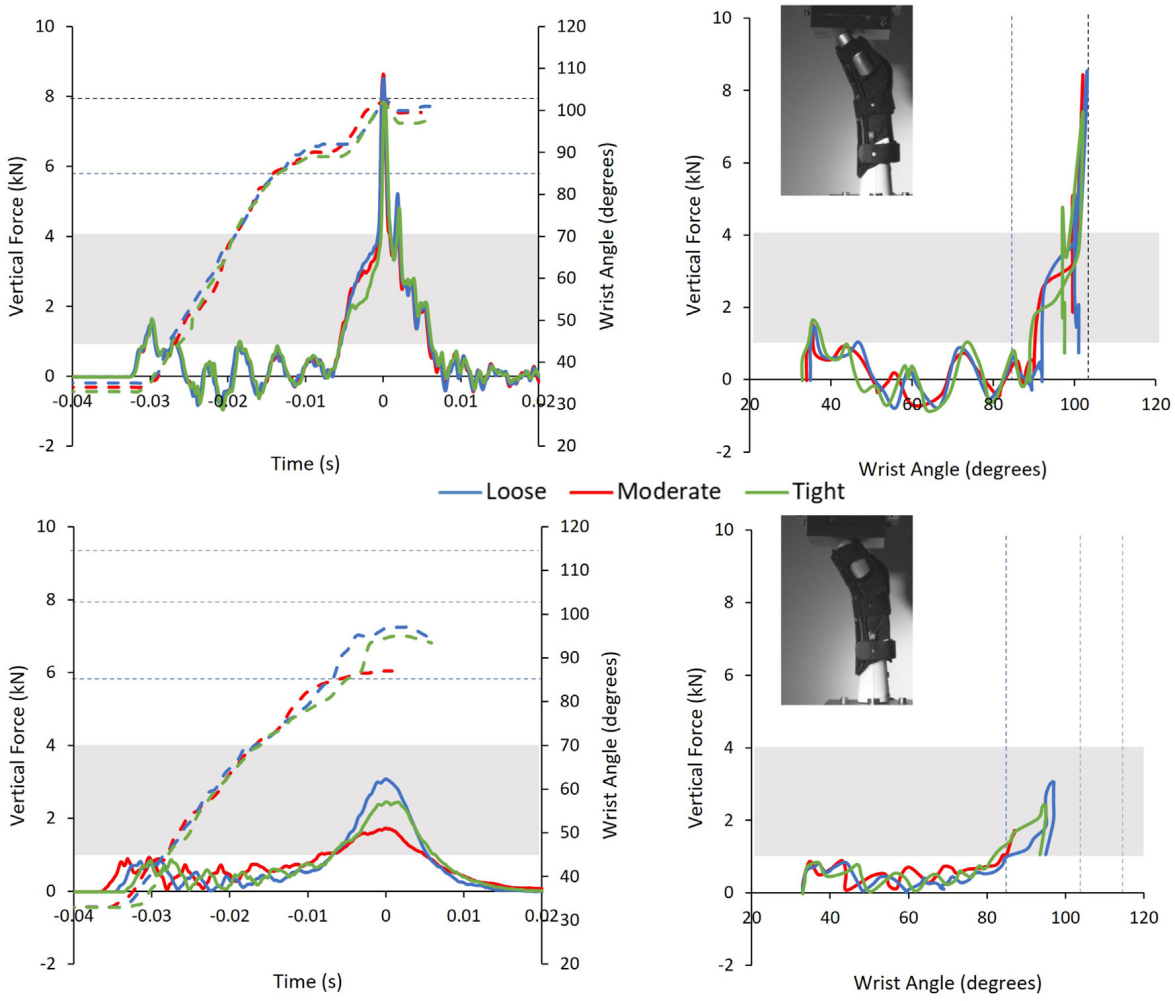
Temporal force and temporal wrist angle (left) and force vs. wrist angle (right) of the first impact of the long protector between strapping conditions on the stiff (top) and compliant surrogate (bottom). Dashed curves indicate the wrist angle. The grey box highlights the cadaver fracture range (∼1–4 ​kN). The dashed straight lines indicate the reported non-injurious maximum wrist extension (85°) and the wrist extension limit of the surrogates (stiff ∼103°, compliant ∼115°).

Protector strapping condition had a significant effect on peak force values, with a large effect size [24], for both surrogates (stiff surrogate p ​= ​0.033, ηp^2^ ​= ​0.25, compliant p ​< ​0.001, ηp^2^ ​= ​0.48; see Online Resource 1 Section 4). When strapped tightly on either surrogate, both protectors tended to have a higher force for a set wrist angle once the palm connected with the impactor (defined by Fig. 3 – Point 3 on graphs and Frame 3), compared to the loose and moderate conditions (Fig. 5, Fig. 6). As strapping tightness increased on the compliant surrogate, the maximum wrist angle tended to decrease for both protectors, and furthermore, mean peak force decreased (Fig. 4, Fig. 5, Fig. 6). In contrast, the stiff surrogate reached its wrist extension limit with either protector at all strapping conditions, and no clear trend of mean peak force was observed (Fig. 4, Fig. 5, Fig. 6).

## 4 Discussion

In agreement with the hypothesis, peak impact forces for both protectors were lower (55–68%, short 3.1 vs. 6.8 ​kN, long 2.7 vs. 8.3 ​kN) when on the compliant surrogate, compared to the stiff surrogate (Fig. 4), and furthermore, the time to reach peak force was typically longer (up to 12%, ∼4 ​ms) (Fig. 3). As with bend testing [8,9], impact results were dependent on protector strapping condition on the surrogate (Fig. 5, Fig. 6). Protectors on the compliant surrogate generally behaved as expected [8,9] and in agreement with the hypothesis; as strapping tightness increased, wrist angle reduced, indicating the protector–surrogate combination was stiffer, and as a result peak force was lower (Fig. 4, Fig. 5, Fig. 6). As such, surrogate design and protector strapping condition can both influence impact test results, as also found for bend testing [[7], [8], [9],^11^]. Therefore, publications reporting on testing of wrist protectors when fitted to a surrogate must clearly describe its design and the strapping conditions, so the results can be objectively compared and reproduced to published studies. Such reporting of limb surrogates and test conditions should make it easier for the standards community to come to a consensus on developing tests for certifying wrist protectors. The surrogates presented here can be reproduced from the stl files provided (see Online Resource 2).

With a protector fitted, the initial spike in force due to the pendulum striking the raised hand of the stiff surrogate [10] was almost twice the value (∼0.9 vs. ∼1.6 ​kN) for the compliant one (Fig. 3, Point 1 on graphs). This lower initial force for the compliant surrogate can be attributed to the silicone on the hand cushioning the impact. As initial contact with the striker caused a clear increase in force with no immediate change in hand angle, its initial position (∼35°) may have been too upright, and the scenario may not have been representative of those where snowboarders injure their wrists. After the initial force spike, both protectors had forces fluctuating between positive and negative values when on the stiff surrogate, whereas they had a steadier positive increase in force when on the compliant one (Fig. 3, Point 2 on graphs). The off-centre impact (i.e., the pendulum first striking the uppermost part of the hand) caused horizontal deflection of the forearm (see Supplementary video), which caused vibrations seen as fluctuating vertical forces, as observed by Adams et al. [10]. The compliant hand was in contact with the pendulum during this period, “anchoring” the surrogate and limiting horizontal deflections and vibrations of the forearm. The compliant surrogate may have “gripped” the protector and impactor due to higher friction, although this was not measured, and could be the subject of future work. When comparing surrogates of varying compliance, the surrogate–protector coefficient of friction should ideally be matched between the different designs and as close as possible to human skin. Giddins and Giddins [25] used online videos to study the position of the upper limbs during unprotected non-injurious skateboarding falls, reporting a maximum wrist angle of 110°. Greenwald et al. [22] reported lower values for non-injurious snowboarding falls (max. mean of <85°), using an instrumented glove. Further work studying snowboarding falls to give better insight into the associated parameters, such as the angle of the wrist throughout impact, would be beneficial to inform testing [22], and the subsequent improvement of ISO 20320:2020.

A rapid increase in force to the peak value was observed when either protector was on the stiff surrogate (Fig. 3, Fig. 5, Fig. 6). This was due to the surrogate reaching its wrist extension limit (103°), with the back of the hand contacting the central core. In contrast, when either protector was on the compliant surrogate, a more gradual increase in force to the peak value was observed, with the wrist extending further with the short one. The more gradual increase to peak force on the compliant surrogate may have been due to the higher maximum possible extension limit (115°), and furthermore, the silicone surrounding the joint compressing (at wrist angles >103°) and acting as a cushion. As such, this raises the question of whether it was the silicone surrounding the joint or the silicone on the palm and forearm of the surrogate that caused lower impact forces for the compliant surrogate. Future work with instrumentation, such as pressure sensors under the protector and strain gauges on splints, combined with finite element modelling [14,26], could give greater insight into how surrogate design influences test results.

There are limitations to this study. The main one being that incorporating silicone into the surrogate influenced the interaction between the impactor and hand and allowed the wrist to extend past its resting limit when loaded. Future work should identify ways to better control such parameters when comparing surrogate designs, to give fairer comparisons. Only two wrist protector designs were tested on one size of surrogate, at just one impact energy at room temperature. Future work could test more protector designs on different sized surrogates, over a range of energies and temperatures related to diverse wrist injury scenarios. Such work could facilitate comparison of results from a complex test like the one present here, with those of the simpler ones within wrist protector standards (ISO 20320:2020 and EN 14120:2003), with a view to finding the simplest protocol that can detect meaningful differences between products and identify those that prevent injury. Such work could inform revisions of wrist protector standards.

As noted by Adams et al. [10], the introduction of ISO 20320:2020 may influence the snowboarding wrist protectors available on the market, and this should be considered when selecting products for testing. Snowboarders tend not to wear wrist protectors [[27], [28], [29], [30], [31]], and subjective factors that could influence user perceptions and uptake, like fit, comfort and perceived performance, could be influenced by changes in material stiffness with temperature. Future work should aim to develop our understanding of the factors that influence wrist protector performance, fit and comfort. Unlike the rigid hemisphere used in both ISO 20320:2020 and EN 14120:2003, the limb surrogates tested here were anthropometric. Further work is needed to determine the suitability of using limb surrogates that are shaped like a human arm in impact tests in wrist protector standards, considering factors such as size, compliance, complexity, test conditions, repeatability, and pass criteria. Such work could help the standards community in reaching a consensus on the most appropriate tests for certifying wrist protectors.

## 5 Conclusion

Surrogate design and strapping condition influenced the performance of two designs of snowboarding wrist protectors in an impact test. Adding a compliant outer layer to an otherwise stiff wrist surrogate reduced the peak impact force and increased the time to peak force of both a short and long protector. For this impact scenario (∼31 ​J impact), the peak force of either protector on the compliant surrogate lay within the cadaver fracture range and exceeded that range when on a comparable stiff surrogate. The wrist extended less, with a lower peak force, when protectors were strapped tighter, with a clear trend observed when the short one was fitted to the compliant surrogate (tight ​> ​moderate ​> ​loose).

## Funding

This research was funded by 10.13039/100010014Manchester Metropolitan University.

## Declaration of competing interest

The authors were involved in the development of ISO 20320:2020.

## Footnotes

1 Supplier website states “The palm skid plate is primarily designed for street sport use” (https://demon-united.com/products/flexmeter-wrist-guard-double-sided-d3o).
