# Supplementary material for: Effect of limb surrogate surface compliance on the impact response of wrist protectors
Source: JSAMS Plus. 2023 Apr 27;2:100023. doi: 10.1016/j.jsampl.2023.100023 (PMC13008448; doi:10.1016/j.jsampl.2023.100023)
Supplement: Multimedia component 3 [file mmc3.pdf]

Online Resource 1 for "Effect of Limb Surrogate Surface Compliance on the Impact Response of Wrist Protectors"

Section 1

It was unfeasible to use the central core from Adams *et al.* [10] and replace the hand and forearm casings to those consisting of the new geometry (medium ISO 20320:2020 surrogate), as the central core would protrude during wrist extension and through the forearm casings. A new central core was therefore made, which housed a toothed timing belt mechanism like the one of Adams *et al.* [10] (Fig. S1 b). The compliant forearm casings (Fig. S1 b part 10) were developed to fit around the central core. Moulds, based on the external geometry of the surrogate, to create a cavity around the hand and forearm casings for the silicone (cured at room temperature) to fill, were created following the same procedure as Leslie *et al.* [8]. The central core was machined (SM3500 CNC milling machine, XYZ Machine Tools Ltd, Devon, UK) from mild steel. The compliant hand core and stiff hand were machined (CNC machine, VF-2, HAAS Automation Ltd, Norwich, UK) from 6061 aluminium. The forearm casings were all laser sintered (PA12, Materialise, Southampton, UK). The hand and forearm moulds were additively manufactured (PLA, Ultimaker, PrintCity, Manchester, UK).

Components masses were as follows: stiff hand 495.5 g, stiff forearm casings (excluding central core) 215.9 g, compliant hand 437.8 g, compliant forearm casings (excluding central core) 113.8 g (Mettler Toledo PE11, Leicester, UK). The compliant parts were lighter because the silicone had a lower density than the aluminium of the hand and the plastic of the casings. The surrogate hands were ~100 g (27 and 44%) heavier than the reported mass of an actual hand (345.3 g) [32[1]], and below the limit of 600 g specified in ISO 20320:2020. As the surrogate forearm was fixed, the mass in comparison to an actual forearm is less important in this impact scenario.

[1] [32] Clarys J.P., Marfell-Jones M.J. Anthropometric prediction of component tissue masses in the minor limb segments of the human body. Hum Biol 1986;1:761–769.

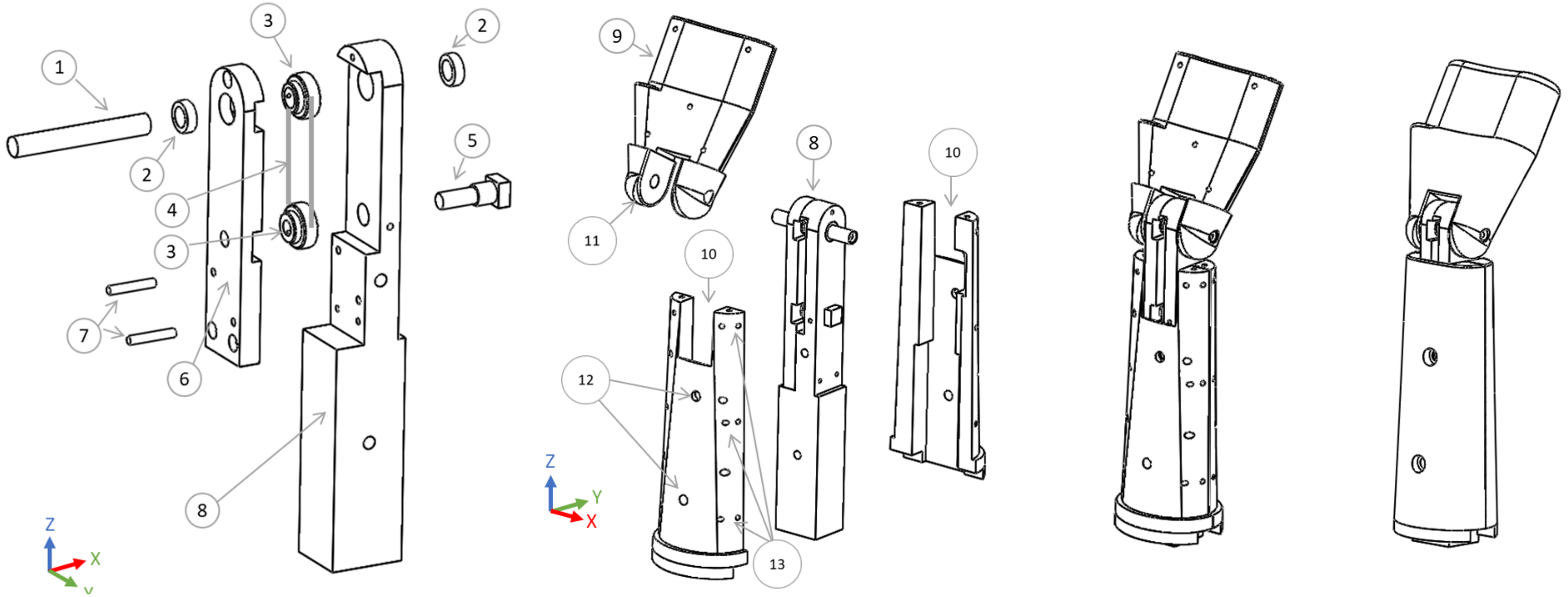

(a) (b) (c) (d)

Fig. S1 – Exploded view of (a) the central core and (b) the compliant surrogate, and assembly of the (c) compliant and (d) stiff surrogate. 1 - shaft, 2 - bearing, 3 - timing pulley, 4 - timing belt, 5 - potentiometer, 6 - central core side part, 7 - dowels, 8 - central core, 9 – hand core, 10 – forearm casings, 11 - grub screw hole, 12 - holes for attachment to central core, 13 - mechanical bonds for silicone. Note the potentiometer was not used here due to malfunction.

Section 2

Temporal wrist angle was measured from the high-speed video footage of the camera viewing side on (Fig. S2). The '*Angle & Angular Speed: 3 point*' function within Phantom Cine Viewer Application was used to measure the angle between three points selected on the video frame: i) a point along the forearm, ii) a point vertically above the previous point and near the wrist joint, iii) a point along the top of the hand. Wrist angles were measured every 10 frames (1 kHz), with extra frames measured at points of interest, corresponding to fluctuations in force. Uncertainty in angle measurements from manual tracking was presumed to be ~1°, based on repeated measurements on an image.

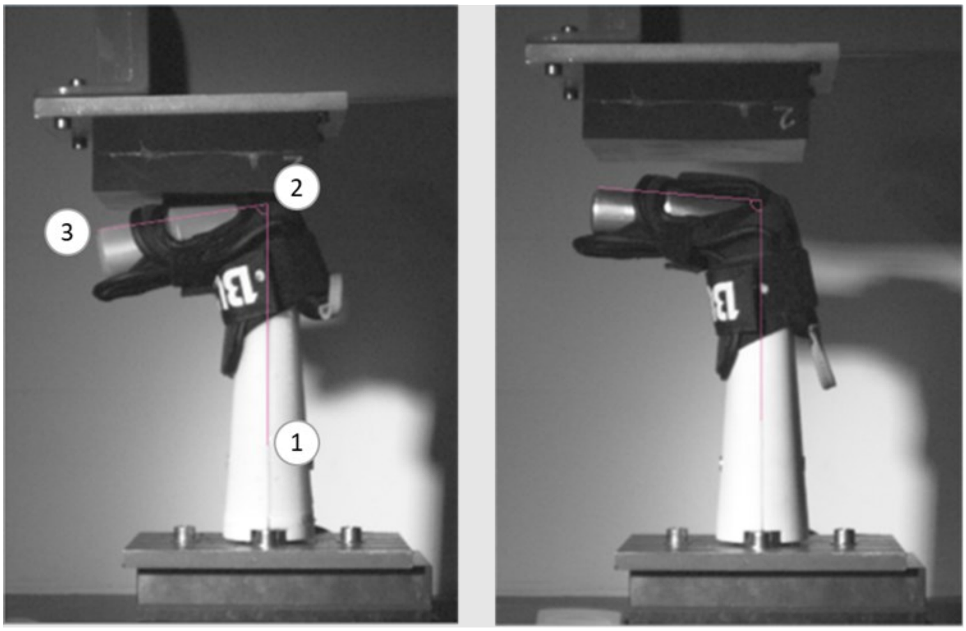

Fig. S2 – Example wrist angle measurement of the short protector on the stiff (left) and compliant (right) surrogate. Pink lines indicates the angle measured. Labels indicate order of points selected.

Section 3

A maximum non-injurious wrist extension angle of 85° was reported from six studies measuring normal ranges of motion of the wrist (Table S3).

Table S3 - Reported maximum non-injurious wrist extension from various studies. Mean ± standard deviation.

	Wrist extension angle (°)	Participants	Reference
	70	-	[16]
	60-85	-	[17]
	67	♂, 28.3 ± 4.9 yrs	[18]
	73	♂, 23.0 ± 4.5 yrs	[19]
	79	♀, 22.4 ± 4.8 yrs	
	76	♂, ≤19 yrs	[20]
	74	♂, >19 yrs	
	72	♂&♀, 24 ± 4 yrs	[21]
Mean ± Standard Deviation	73 ± 6.7		
Range	60 to 85		

Section 4

The general linear model univariate analysis showed most main effects were significant ($p < 0.05$) with a large effect size ($\eta p^2 > 0.01$ small effect, $\eta p^2 > 0.06$ medium effect, $\eta p^2 > 0.14$ large effect [24]) (Table S4). Protector style (short, long) had a larger effect on the stiff surrogate than the compliant one ($\eta p^2 = 0.84$ vs. 0.36), and the strapping condition (loose, moderate, tight) had a larger effect on the compliant surrogate than the stiff surrogate ($\eta p^2 = 0.48$ vs. 0.25). Protector condition (new, used) had the smallest effect on both surrogates ($\eta p^2 = 0.24$ (stiff), < 0.01 (compliant)), and was not significant on the compliant surrogate.

Table S4 – General linear model univariate between subject effects for the stiff and compliant surrogate (* indicates significant result).

Surrogate	Source	df_1	df_2	F	p-value	Partial Eta Squared (ηp^2)
**Stiff**	Protector style	1	24	126.82	< 0.001*	0.84 (large effect)
	Strapping condition	2	24	3.93	0.033*	0.25 (large effect)
	Protector condition	1	24	7.44	0.012*	0.24 (large effect)
**Compliant**	Protector style	1	24	13.67	0.001*	0.36 (large effect)
	Strapping condition	2	24	11.14	< 0.001*	0.48 (large effect)
	Protector condition	1	24	0.10	0.753	< 0.01 (small effect)
